# Circular RNA circTRPS1-2 inhibits the proliferation and migration of esophageal squamous cell carcinoma by reducing the production of ribosomes

**DOI:** 10.1038/s41420-023-01300-9

**Published:** 2023-01-12

**Authors:** Renchang Zhao, Pengxiang Chen, Chenghao Qu, Jinghui Liang, Yulan Cheng, Zhenguo Sun, Hui Tian

**Affiliations:** 1 Department of Thoracic Surgery, Qilu Hospital of Shandong University, Jinan, Shandong 250012 China; 2 Laboratory of Basic Medical Sciences, Qilu Hospital of Shandong University, Jinan, Shandong 250012 China; 3 Advanced Medical Research Institute/Translational Medicine Core Facility of Advanced Medical Research Institute, Shandong University, Jinan, Shandong 250012 China; 4 Department of Radiation Oncology, Qilu Hospital of Shandong University, Jinan, Shandong 250012 China; 5 Program in Molecular and Cellular Oncogenesis, The Wistar Institute, Philadelphia, PA USA; 6 Department of Medicine/GI Division, Johns Hopkins University School of Medicine and Sidney Kimmel Comprehensive Cancer Center, Baltimore, MD 21287 USA

**Keywords:** Prognostic markers, Cancer genomics

## Abstract

Circular RNAs play important roles in many cancers, including esophageal squamous cell carcinoma (ESCC), but the precise functions of most circular RNAs are poorly understood. Here we detected significant downregulation of circTRPS1-2 in ESCC based on high-throughput sequencing of three pairs of ESCC tissue and adjacent normal tissue, followed by PCR validation with another 30 tissue pairs. Patients with ESCC whose circTRPS1-2 expression was below the median level for the sample showed significantly shorter median overall survival (13 months) than patients whose circTRPS1-2 expression was above the median (36 months). Overexpressing circTRPS1-2 in the human ESCC cell lines K150 and E109, which express low endogenous levels of circTRPS1-2, inhibited cell proliferation and migration. Conversely, knocking down circTRPS1-2 using short interfering RNA promoted cell proliferation and migration. Similar results were observed in mice bearing K150 xenografts in which circTRPS1-2 was overexpressed or knocked down. Several ribosomal proteins co-immunoprecipitated with circTRPS1-2 from K150 cells in culture, and K150 cells overexpressing circTRPS1-2 showed reduced numbers of ribosomes by A260 absorbance measure and electron microscopy. Our results suggest that circTRPS1-2 can inhibit ESCC proliferation and migration by reducing the production of ribosomes, establishing its potential usefulness in ESCC treatment and prediction of prognosis.

## Background

Esophageal squamous cell carcinoma (ESCC) is globally the most common form of esophageal cancer, which ranks seventh among cancers in terms of morbidity and sixth in terms of mortality [1]. More than half of ESCC cases worldwide occur in China [2]. Despite advances in therapeutic strategies, the late onset of symptoms and tumor heterogeneity in ESCC mean poor prognosis for most patients [3, 4]. Therefore, understanding how the disease progresses and identifying reliable prognostic markers may improve treatment and monitoring, translating to better prognosis.

Circular RNAs (circRNAs) are closely associated with the development of various human cancers, including ESCC [5, 6]. CircRNAs are circular, noncoding RNAs that arise through back-splicing of precursor mRNAs [7]. They can “sponge” microRNAs [8, 9] or interact with RNA-binding proteins [10] to regulate cell proliferation, differentiation, migration, and apoptosis, thereby influencing tumor progression [11]. One class of RNA-binding proteins is ribosomal proteins, which form part of ribosomes.

Here we performed high-throughput sequencing of three matched pairs of ESCC and adjacent normal tissue, identifying a new functional circRNA, hsa_circ_0085362, which we designated circTRPS1-2. We found that circTRPS1-2 was downregulated in ESCC and that low expression correlated with worse prognosis. We further found that overexpressing circTRPS1-2 inhibited the proliferation and metastasis of ESCC cells in culture and in xenograft models. Lastly, we identified interactions between circTRPS1-2 and the ribosomal proteins S4 protein (X-linked), S8, S24, L7 and L11; and we found that overexpressing the circRNA reduced the number of ribosomes in the cell. Our results indicate that circTRPS1-2 may be a novel prognostic marker and therapeutic target in ESCC.

## Materials and methods

### ESCC sample collection

Formaldehyde-fixed, paraffin-embedded samples of ESCC tissues and adjacent normal esophageal tissues were obtained from the Pathology Department of Qilu Hospital at Shandong University (Jinan, China). The samples had been obtained in 2018 from middle-aged and elderly patients who underwent radical resection for advanced ESCC without neoadjuvant therapy in the Thoracic Surgery Department of Qilu Hospital. The adjacent normal tissues were taken more than 5 cm away from the tumor margin. Three matched pairs of tissues were used for high-throughput sequencing, while another 30 matched pairs were used for validation by quantitative real-time PCR (qRT–PCR, see below). Patients were followed up from surgery until December 31, 2021. The use of the clinical samples in this study was approved by the Ethics Committee of Qilu Hospital, which waived the requirement for informed consent because patients, at the time of treatment, provided written consent for their anonymized medical data to be analyzed and published for research purposes.

### High-throughput sequencing of circRNAs

High-throughput sequencing and subsequent bioinformatic analysis of circRNAs were performed by Cloud-Seq Biotech (Shanghai, China) on three pairs of ESCC and adjacent normal tissues. Briefly, total RNA was treated using the Ribo-Zero rRNA Removal Kit (Illumina, CA, USA) to remove ribosomal RNA, and the resulting RNA was used to construct RNA libraries using the TruSeq Stranded Total RNA Library Prep Kit (Illumina) according to the manufacturer’s instructions. Libraries were quality-controlled and quantified using the BioAnalyzer 2100 System (Agilent Technologies, CA, USA), then sequenced (150 cycles) on the HiSeq 4000 platform (Illumina) according to the manufacturer’s instructions. The quality of paired-end reads was checked in terms of the Q30 metric on the HiSeq 4000 platform. High-quality reads were trimmed using Cutadapt software (v1.9.3) [12] and aligned to the reference genome or transcriptome using STAR software (v2.5.1b) [13]. CircRNAs were identified using DCC software (v0.4.4) [14] and annotated using the circBase [15] and circ2Traits [16] databases. Data on the expression of circRNAs were normalized and expression differences between cancerous and healthy tissue analyzed using EdgeR software (v3.16.5) [17]. Expression differences were validated by qRT–PCR using another 30 pairs of tissues, which also allowed verification of the specificity of the primers used to detect circRNAs. Tissues were ranked according to low or high expression of certain circRNAs, and the 15 samples showing the highest expression of a given circRNA were categorized as “high expression” for that circRNA, while the 15 samples showing the lowest expression were categorized as “low expression”. Overall survival was compared between the two sets of samples.

### Cell culture

The ESCC cell lines KYSE150 (hereafter K150) and ECA109 (hereafter E109) were acquired from the China Center for Type Culture Collection (Wuhan, China). Healthy human esophageal epithelial cells (HEECs) were obtained from Dr. Peichao Li of the Second Hospital of Shandong University (Jinan, China). All cell lines used were authenticated by short tandem repeat profiling. All cell lines were maintained in a humidified incubator at 37 °C in a 5% CO_2_ atmosphere. HEECs were cultured in Dulbecco’s modified Eagle medium (Gibco, CA, USA), while cancer cells were cultured in RPMI 1640 (Gibco). In all cases, the medium was supplemented with 10% fetal bovine serum (Gibco) as well as 100 U/ml penicillin G and streptomycin (Solarbio, Beijing, China).

### RNA extraction and qRT–PCR

Total RNA was extracted from tissues and cells using the MiniBEST Universal RNA Extraction Kit (Takara, Dalian, China). Nuclear and cytoplasmic RNA was extracted using the Cytoplasmic & Nuclear RNA Purification Kit (Norgen Biotek, Thorold, ON, Canada). Total RNA (1 µg) was incubated with 3 U of RNase R (RiboBio, Guangzhou, China) at 37 °C for 30 min, then converted to cDNA using the PrimeScript RT Master Mix (Takara). qRT–PCR was performed using the SYBR Premix Ex Tap II kit (Takara) in a LightCycler 480 system (Roche Diagnostics, Rotkreuz, Switzerland). Cycle thresholds were determined using the manufacturer’s software; β-actin, GAPDH and U1 snRNA served as reference genes. Accurate Biology (Changsha, China) designed and synthesized primers for amplification of all circRNAs and of mRNAs encoding trichorhinophalangeal syndrome 1 (TRPS1), GAPDH and β-actin (Table S1).

### Fluorescence in situ hybridization (FISH)

Cy3-labelled circTRPS1-2 probes (Table S1) were designed by GenePharma (Shanghai, China) and used for FISH in a commercial kit (GenePharma). K150 and E109 cells (1 × 10^4^) were seeded onto coverslips in 48-well plates, incubated overnight, then fixed with 4% paraformaldehyde for 15 min at room temperature. Cells were treated with 0.1% Buffer A, then with 2× Buffer C. Prewarmed Buffer E was mixed with 0.5 µL of probes, the mixture was denatured at 73 °C for 5 min, then 100 µL of the mixture containing probes at 2 µM was added to each well. Hybridization was allowed to proceed overnight in an incubator, then wells were washed with Buffer F followed by Buffer C. Finally, coverslips were stained with DAPI, mounted with antiquenching agent (Solarbio), and observed under a fluorescence microscope (Olympus BX53, Tokyo, Japan).

### BaseScope assay

The BaseScope™ RED Kit 2 (Advanced Cell Diagnostics, Hayward CA, USA) was used. Slides were baked in a dry oven for 1 h at 60 °C. Formaldehyde-fixed, paraffin-embedded tissue sections were dewaxed for approximately 20 min and incubated in H_2_O_2_ for 10 min to quench endogenous peroxidase activity. Antigens were retrieved for 15 min, RNAScope protease reagent was applied for 15 min, then probes (catalog no. 1038201-C1, Advanced Cell Diagnostics) were hybridized for 2 h. The AMP1-8 were applied in sequence according to the manufacturer’s instructions, then slides were dried, mounted and observed under a microscope (Olympus CKX31). Cells were considered positive if they contained a visible red dot or cluster at a magnification of 40 ×. Scoring criteria: 0, positive cells < 1 per 20 cells; 1, 1 ≤ positive cells < 2 per 20 cells; 2, 2 ≤ positive cells < 3 per 20 cells; 3, 3 ≤ positive cells < 4 per 20 cells; 4, positive cells ≥ 4 per 20 cells.

### Overexpression and silencing of circTRPS1-2

A plasmid and lentiviral vector to overexpress circTRPS1-2 was generated by subcloning the circTRPS1-2 sequence (circBase: hsa_circ_0085362) into GV486 and GV348 vector, respectively. CircTRPS1-2 was knocked down in K150 and E109 using short interfering RNAs (siRNAs), which were synthesized by RiboBio. In parallel, negative control siRNAs were used to confirm the specificity of the results. The most effective siRNA was used to construct a short hairpin RNA (shRNA) in a GV493 lentiviral vector. The overexpression plasmid as well as lentiviral vectors encoding the human circTRPS1-2 sequence, the shRNA targeting circTRPS1-2 or empty vector as negative control (Table S1) were synthesized by GeneChem (Shanghai, China). Transfection was performed using jetPRIME (Polyplus Transfection, Strasburg, France).

### Cell counting Kit-8 assays

Proliferation of ESCC cell lines was assessed using an Enhanced CCK-8 Kit (Bioss, Beijing, China). K150 and E109 cells (2 × 10^3^ per well) were seeded into 96-well plates in triplicate, incubated for 24 h. The reagent was added and incubated for 2 hours according to the manufacturer’s instructions. Absorbance at 450 nm was measured using a spectrophotometer (Synergy4, BioTek, Winooski, VT, USA).

### EdU incorporation assay

K150 and E109 cells (2 × 10^5^ per well) were seeded into 96-well plates in triplicate, incubated for 24 h, then stained with the Cell-Light™ EdU 156 Imaging Detection Kit (RiboBio) following the manufacturer’s instructions. Cultures were observed under a fluorescence microscope (Olympus BX53).

### Colony formation

A total of 2 × 10^3^ cells per well were seeded into six-well plates in triplicate, incubated for 10–12 days, fixed with methanol for 20 min, and stained with crystal violet. Colonies larger than 50 cells were counted.

### Transwell migration

A total of 1 × 10^5^ cells in 200 µL of serum-free medium were seeded into the upper chambers of a 24-well Transwell system (Corning, New York, USA). Medium (800 µL) containing 20% fetal bovine serum was added to the lower chambers. After incubation for 24 h, the membranes in the Transwell chambers were treated with methyl alcohol and crystal violet. Cells were counted under a microscope (Olympus CKX31).

### Subcutaneous tumorigenesis in vivo

Four-week-old female BALB/c nude mice were purchased from Jiangsu GemPharmatech Biotechnology (Nanjing, China) and maintained in a qualified biosafety level 2 animal laboratory. Mice were randomly assigned into each group using random number table. K150 cells (5 × 10^6^ per animal) infected with recombinant lentivirus were injected subcutaneously into the right axillae of the mice for the tumorigenesis assay. The long and short diameters of the tumors were measured at 2-day intervals starting approximately 1 week after injection, when nodules first became visible. Tumor volumes were calculated using the formula: Volume = (short diameter)^2^ × (long diameter) × 0.5 [18]. At 28 days after injection, subcutaneous tumors were excised and analyzed as described below.

### Tumor metastasis

Four-week-old female BALB/c nude mice were purchased from Jiangsu GemPharmatech Biotechnology and maintained in the animal laboratory for 2 weeks. Mice were randomly assigned into each group using random number table. K150 cells (1 × 10^6^ per animal) infected with recombinant lentivirus were injected into the mice *via* the caudal vein, and metastasis was assessed 2 months later. Only superficial metastasis was assessed because of the weak penetration of green fluorescence; for this purpose, the Spectrum 200 in vivo imaging system (PerkinElmer, Waltham, MA, USA) was used in circTRPS1-2 knockdown group and control. In circTRPS1-2 overexpressing group, superficial and lung metastases had to be counted individually rather than detected with fluorescence because we were unable to insert the gene for green fluorescent protein into the lentiviral vector. Lungs showing metastasis were excised for further analysis (see below). Mice were euthanized and solid tumors were excised. Tumor weight never constituted more than 10 percent of total body weight. All animal experiments were approved by the Institutional Animal Care and Use Committee of Qilu Hospital of Shandong University and were performed according to institutional guidelines.

### RNA pulldown

Biotin-labelled sense and antisense RNA probes were synthesized by GenePharma (Table S1) and used as “bait” in RNA pulldown assays with the Pierce Magnetic RNA–Protein Pull-Down Kit (ThermoFisher Scientific, Waltham, MA, USA). According to the manufacturer’s instructions, K150 cells (1 × 10^7^ per sample) were prepared using 500 µl of lysis buffer (Thermo Fisher Scientific), and total protein concentration was measured using a BCA assay kit (Beyotime). After a prewash, streptavidin-conjugated magnetic beads (50 µl) were incubated with 50 pmol of labelled RNA, and the resulting RNA-bead complexes were incubated with total lysates (200 µg). Beads were washed, then RNA-binding proteins were eluted and analyzed as described below.

### Mass spectrometry

K150 cells (1 × 10^7^ per sample) were treated using a Total Protein Extraction Kit (BestBio, Shanghai, China), and protein concentration was measured using a BCA assay (Beyotime). Protein (100 µg in 10 µl) was incubated with 50 µl of denaturation buffer [0.5 M Tris-HCl (pH 8.1 ± 0.1), 2.75 mM EDTA, 6 M guanidine-HCl]. Then 30 µl of 1 M DTT was added, the mixture was incubated at 37 °C for 2 h, 50 µl of 1 M iodoacetamide was added, and the mixture was incubated in the dark at room temperature for 1 h. Samples were concentrated by centrifugation on YM-3 ultrafiltration filters (Millipore, MA, USA), then washed four times with HPLC water. Samples were incubated at 37 °C overnight with 4 µg of trypsin, and purified through C18 pipette tip filters (ZipTip, Millipore). Proteins were identified and quantified by liquid chromatography and mass spectrometry on an Orbitrap Elite system (ThermoFisher) at Shandong University.

### RNA immunoprecipitation

K150 cells (1 × 10^7^ per sample) were lysed and mixed with magnetic beads conjugated to antibodies using the Magna RNA-Binding Protein Immunoprecipitation Kit (Millipore) following the manufacturer’s instructions. RNA that co-immunoprecipitated with antibodies was eluted by treating the beads with proteinase K, then analyzed by qRT–PCR.

### Western blotting

Samples were boiled, fractionated on SDS-polyacrylamide gels and transferred to polyvinylidene fluoride membranes (Millipore). Membranes were blocked with NcmBlot blocking buffer (New Cell & Molecular Biotech, Suzhou, China), incubated with primary antibodies at 4 °C overnight, then incubated with secondary antibodies at room temperature for 1 h, and finally visualized using the Immobilon Western Chemiluminescence kit (Millipore).

### Antibodies

Antibodies against the following ribosomal proteins were used for Western blotting and immunofluorescence: L7 (Huabio, Hangzhou, China), L11 (Huabio), S4X (Huabio), S8 (Huabio), and S24 (Proteintech, Wuhan, China). Antibodies were also used against IgG (ThermoFisher) and β-actin (Abcam, Cambridge, UK). Goat secondary antibodies against rabbit or mouse primary antibodies (Huabio) were used at a dilution of 1:5000.

### Immunofluorescence

K150 cells (1 × 10^4^) were seeded onto coverslips in 48-well plates, incubated overnight, fixed with methanol for 20 min, permeabilized with 0.2% Triton X-100 in phosphate-buffered saline (PBS) for 20 min, blocked at room temperature for 1 h with 5% goat serum (Zhong Shan Golden Bridge Biological Technology, Beijing, China), incubated at 4 °C overnight with antibodies against ribosomal proteins, and finally incubated at 37 °C for 1 h with secondary antibody conjugated to Alexa Fluor 488 (1:500; GeneCopoeia, Rockville, MD, USA) or DyLight 594 (1:500; Abbkine Scientific, Wuhan, China). Coverslips were mounted with an antifade solution containing DAPI (Solarbio).

### Electron microscopy

K150 cells (5 × 10^6^ per sample) were fixed with glutaraldehyde at 4 °C for more than 2 h, washed with Milloning phosphate buffer at 4 °C, postfixed with osmic acid for 2 h, dehydrated with ethanol, and impregnated with epoxy resin and hardener. Samples were sliced, stained with uranyl acetate, and observed on a High-Tech transmission electron microscope (Hitachi, Japan).

### Ribosome extraction

K150 cells (1 × 10^7^ per sample) were treated using the Ribosome Extraction Kit (BestBio) according to the manufacturer’s instructions. Cells were centrifuged at 1000 g at 4 °C for 5 min, and the pellet was washed twice with cold PBS, then incubated with 1 ml of cold Reagent A on ice for 10 min and homogenized with 20–30 strokes in a Dounce homogenizer. The homogenate was centrifuged at 1000 g for 5 min at 4 °C, the supernatant was transferred to a fresh tube and centrifuged at 4 °C at 20,000 g for 10 min, and the resulting supernatant was centrifuged at 4 °C and 120,000 g for 60 min. The pellet from this centrifugation was resuspended in 400 μl of cold Reagent B, then centrifuged again at 4 °C and 120,000 g for 60 min. The resulting pellet was resuspended in 250 μl Ribosome Preservation Solution, and the absorbance at 260 nm was measured using the NanoDrop™ One Microvolume Spectrophotometer (ThermoFisher).

### Bioinformatic analysis

Metascape [19] was used to perform Gene Ontology enrichment analysis, Reactome Pathway analysis and Kyoto Encyclopedia of Genes and Genomes pathway enrichment analysis.

### Statistical analysis

Statistical analyses were performed using Prism 8.0.1 (GraphPad Software, San Diego, CA, USA) and SPSS 25.0 (IBM, SPSS, Chicago, IL, USA). The differences of clinicopathological features between groups were analyzed using Fisher’s exact test. Other differences between groups were assessed for significance using student’s *t* test or ANOVA as appropriate. Overall survival was analyzed using the Kaplan–Meier method, and survival curves were compared using the log-rank test. No blinding was used. Sample sizes were chosen without pre-specified effect sizes. Data were shown as mean ± standard deviation (SD). Differences that were associated with *P* < 0.05 were considered statistically significant.

## Results

### Expression of circTRPS1-2 in ESCC tissues

**Fig. 1:**
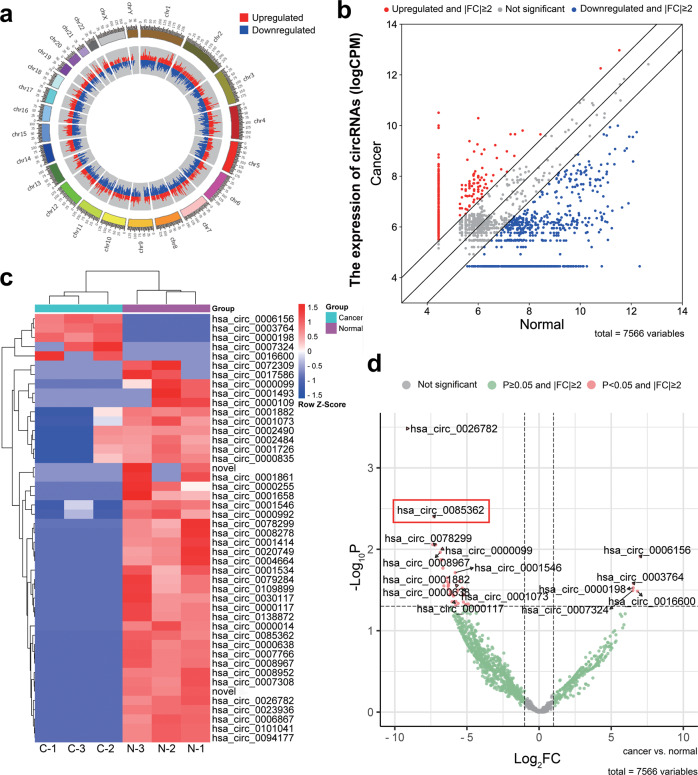
Identification of circTRPS1-2 through high-throughput sequencing of ESCC tissues. **a** Circos plot showing the chromosome positions of all circRNAs identified in ESCC tissues. **b** Differences in expression of circRNAs between ESCC and adjacent normal esophageal tissues. **c** Heatmap showing differences in expression of 41 circRNAs downregulated in ESCC tissues and 5 circRNAs upregulated in ESCC tissues. Differences are expressed as log (CPM) as calculated by EdgeR. **d** Volcano plot showing 10 circRNAs downregulated in ESCC tissues and 5 upregulated circRNAs. The circRNA hsa_circ_0085362 is marked. Grey indicates no significant difference; green, *P* ≥ 0.05 and | FC | ≥ 2; and red, *P* < 0.05 and | FC | ≥ 2, which served as the definition of differential circRNA expression. C or Cancer, ESCC tissue; CPM, counts per million; ESCC, esophageal squamous cell carcinoma; FC, fold change; N or Normal, adjacent normal esophageal tissue.

High-throughput sequencing of three matched pairs of ESCC and adjacent normal tissue identified 2685 circRNAs that were upregulated in the cancer and 4881 that were downregulated (Fig. 1a, b). Applying cut-off criteria of ∣fold change ∣ ≥ 2 and *P* < 0.05 identified a subset of five upregulated circRNAs (hsa_circ_0006156, hsa_circ_0003764, hsa_circ_0000198, hsa_circ_0007324, and hsa_circ_0016600) and 41 downregulated circRNAs (Fig. 1c). Using an independent set of 30 pairs of ESCC and adjacent normal tissues, we validated the five upregulated circRNAs and the following 10 most downregulated circRNAs (Fig. 1d): hsa_circ_0026782, hsa_circ_0085362 (circTRPS1-2), hsa_circ_0078299, hsa_circ_0000099, hsa_circ_0008967, hsa_circ_0001546, hsa_circ_0001882, hsa_circ_0001073, hsa_circ_0000638, and hsa_circ_0000117. CircRNAs that were detected with primers showing weak specificity were excluded. In the end, we decided to focus on the most dysregulated circRNA, circTRPS1-2, for further study (Table 1).

**Table 1:** *P* values associated with the difference in expression of certain circRNAs between ESCC and adjacent normal tissue in 30 matched pairs of tissues.

CircRNA	*P* value
hsa_circ_0006156	0.0281^*^
hsa_circ_0026782	0.026^*^
hsa_circ_0085362	<0.0001^***^
hsa_circ_0078299	0.0002^***^
hsa_circ_0000099	<0.0001^***^
hsa_circ_0008967	0.0237^*^

*P < 0.05, **P < 0.01, ***P < 0.001.

### Downregulation and subcellular localization of circTRPS1-2 in ESCC cell lines

**Fig. 2:**
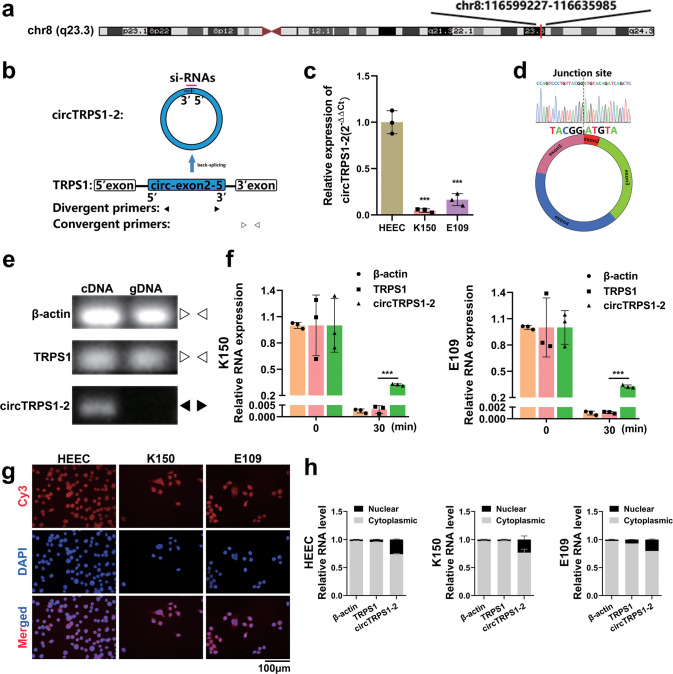
Characterization and subcellular localization of circTRPS1-2 in ESCC tissues. **a** Chromosomal location of circTRPS1-2, as determined using the UCSC Genome Browser. **b** Schematic of the biogenesis of circTRPS1-2. Locations of the forward and reverse primers for amplification of circTRPS1-2 and linear TRPS1, as well as the locations of the siRNAs targeting circTRPS1-2. **c** Expression of circTRPS1-2 in HEECs and two ESCC cell lines. Results are from three independent experiments. **d** Sanger sequencing confirming the back-spliced sequence. **e** Detection of circTRPS1-2 in cDNA but not genomic DNA, based on agarose gel electrophoresis. **f** Levels of circTRPS1-2 and linear TRPS1 in total RNA from K150 or E109 cells before (0 min) and after (30 min) RNase R treatment, as determined by qRT–PCR. Results are from three independent experiments. **g** FISH to determine the subcellular localization of circTRPS1-2 in HEECs and ESCC cells. Magnification, 400×. Scale bar, 100 µm. **h** Nuclear-cytoplasmic fractionation to determine the subcellular localization of circTRPS1-2. Results are from three independent experiments. β-actin was the reference gene. Data are mean ± SD. *P < 0.05, **P < 0.01, ***P < 0.001. FISH, fluorescence in situ hybridization; HEEC, healthy human esophageal epithelial cells; qRT–PCR, quantitative real-time PCR; siRNA, short interfering RNA.

CircTRPS1-2 is located on chromosome 8:116599227-116635985 according to the UCSC Genome Browser (GRCh37/hg19) (Fig. 2a). Containing 2821 bp, it is predicted to arise through back-splicing of exon 2 to exon 5 of the TRPS1 gene (Fig. 2b). We found that circTRPS1-2 was downregulated in both K150 and E109 cell lines relative to HEECs (Fig. 2c). The back-splice junction was validated by Sanger sequencing of qRT–PCR products (Fig. 2d). Divergent primers were able to amplify circTRPS1-2 from cDNA but not from genomic DNA, whereas convergent primers amplified linear TRPS1 from both cDNA and genomic DNA (Fig. 2e). These results support the idea that circTRPS1-2 is produced *via* back-splicing rather than trans-splicing or genomic rearrangement. The circular structure of CircTRPS1-2 was confirmed by its resistance to digestion by RNase R (Fig. 2f). CircTRPS1-2 localized primarily to the cytoplasm and nucleus, based on FISH (Fig. 2g) and nuclear-cytoplasmic fractionation (Fig. 2h).

### Downregulation of circTRPS1-2 in ESCC tumors and correlation with poor prognosis

**Fig. 3:**
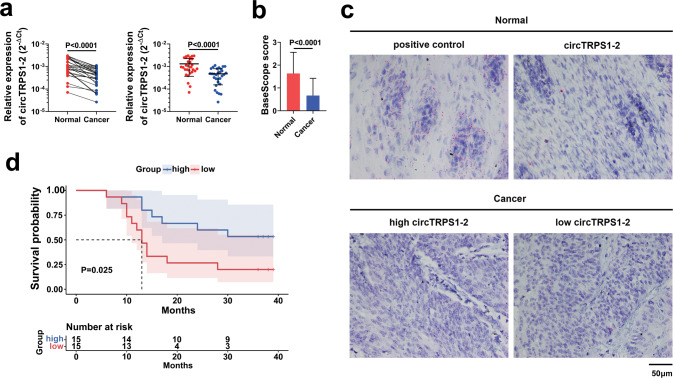
Downregulation of circTRPS1-2 in ESCC tissues and positive correlation between its expression and prognosis. **a** Expression of circTRPS1-2 was significantly lower in ESCC tissues than in adjacent normal tissues. β-actin served as reference genes. Results are from three independent experiments. **b** The results of BaseScope assay showed that the circTRPS1-2 signals in ESCC tissues were significantly less than that in adjacent normal tissues. Data are mean ± SD (*n* = 30). **c** Representative images from the BaseScope assay of ESCC tissues and adjacent normal tissues. Cells contained a visible red dot or cluster were considered positive. positive control, human PPIB gene probes; circTRPS1-2, hsa_circ_0085362 probes. Magnification, 400×. Scale bar, 50 µm. **d** Overall survival time was significantly longer among patients expressing high circTRPS1-2 than among patients expressing low circTRPS1-2.

In a set of 30 matched ESCC and adjacent normal tissue from patients, we found that circTRPS1-2 was significantly downregulated in tumors (Fig. 3a). Similar results were obtained in the BaseScope assay, and the circular RNA localized mainly to the cytoplasm and nucleus (Fig. 3b, c). Its expression tended to correlate with T and TNM stages, although these correlations were nonsignificant (Table S2). Patients with circTRPS1-2 expression below the median level in the set of 30 patients showed significantly lower 3-year overall survival than those with expression above the median (*P* = 0.025; Fig. 3d). These results suggest that circTRPS1-2 may act as a tumor suppressor against ESCC, and that its low expression predicts poor prognosis.

### CircTRPS1-2 suppresses the proliferation and migration of ESCC cell lines

**Fig. 4:**
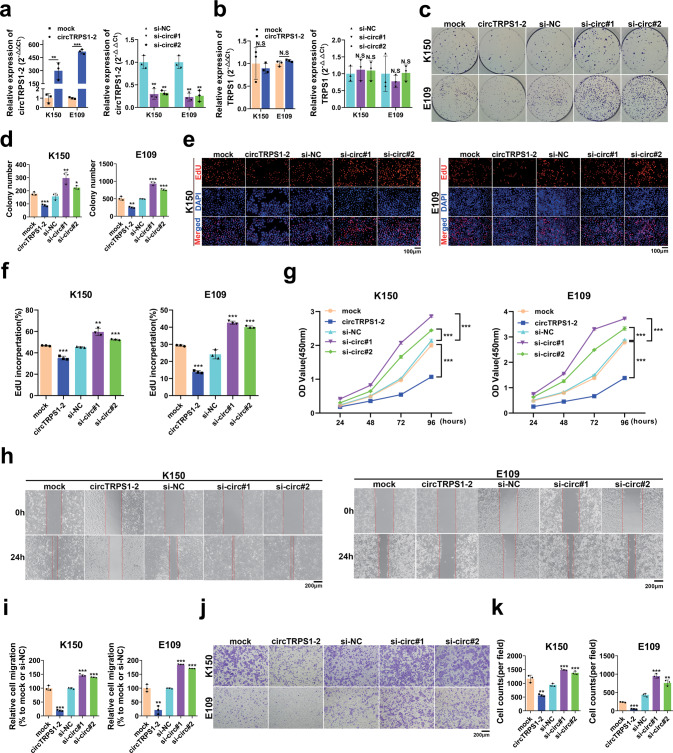
Suppression of ESCC cell proliferation and migration by circTRPS1-2. **a**, **b** Confirmation of circTRPS1-2 overexpression or knockdown in ESCC cells, based on qRT–PCR. Expression of linear TRPS1 was not affected. β-actin was the reference gene. **c**, **d** Colony formation assay to assess the survival of ESCC cells over- or underexpressing circTRPS1-2. **e**, **f** EdU incorporation assay to evaluate the proliferation of ESCC cells. Magnification, 200×. Scale bar, 100 µm. **g** Cell counting Kit-8 assay to evaluate cell viability. **h**, **i** Wound healing assay to evaluate the migration of ESCC cells. Magnification, 100×. Scale bar, 200 µm. **j**, **k** Transwell assay to measure ESCC cell migration. Magnification, 100×. Scale bar, 200 µm. Data are mean ± SD (*n* = 3 independent experiments). *P < 0.05, **P < 0.01, ***P < 0.001. circTRPS1-2, circTRPS1-2 overexpression plasmid; mock, negative control plasmid; si-NC, negative control siRNA; si-circ#1 or 2, circTRPS1-2 siRNA#1 or 2, respectively.

To investigate the biological function of circTRPS1-2 in ESCC cells, two ESCC cell lines were transfected either with a plasmid to overexpress circTRPS1-2 or siRNAs to knock it down (Fig. 4a). These transfections did not significantly affect expression of linear TRPS1 (Fig. 4b). Overexpression of circTRPS1-2 significantly reduced the viability and proliferation of ESCC cells based on assays of colony formation (Fig. 4c, d), EdU incorporation (Fig. 4e, f) and Cell counting Kit-8 (Fig. 4g). It also significantly decreased the migratory ability of ESCC cells based on assays of wound healing (Fig. 4h, i) and transwell migration (Fig. 4j, k). Knockdown of circTRPS1-2 had the opposite effects.

### CircTRPS1-2 inhibits tumorigenesis and metastasis in vivo

**Fig. 5:**
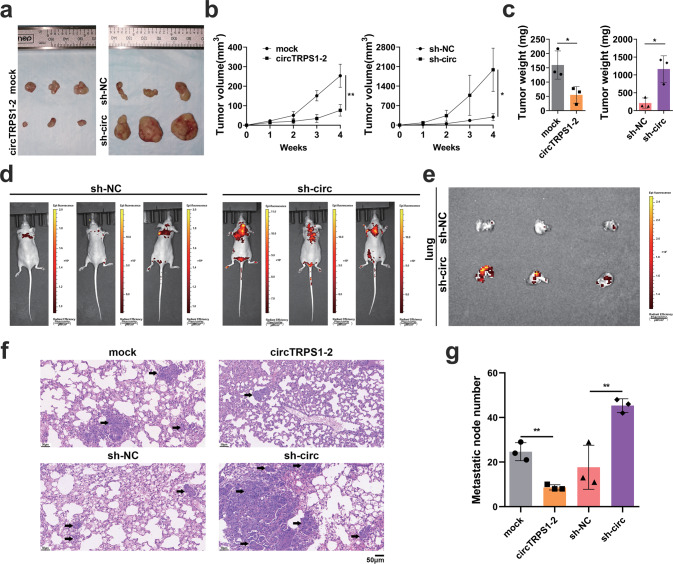
CircTRPS1-2 inhibits the tumorigenesis and metastasis of ESCC in vivo. **a** Representative images of subcutaneous tumors from each group. **b** Growth curves based on weekly measurements of tumor volumes. **c** Tumor weights. **d** Numbers of superficial metastatic nodules in circTRPS1-2 knockdown group, based on IVIS 200 Spectrum analysis. **e** Representative lung metastases in circTRPS1-2 knockdown group, based on IVIS 200 Spectrum analysis. **f** Hematoxylin-eosin staining of lung metastatic nodules. Black arrows indicate metastatic nodule. Magnification, 200×. Scale bar, 50 µm. **g** Total number of metastatic nodules. Data are mean ± SD (*n* = 3 biologically independent samples). *P < 0.05, **P < 0.01. circTRPS1-2, circTRPS1-2 overexpression lentivirus; IVIS, in vivo imaging system; mock and sh-NC, negative control lentivirus; sh-circ, circTRPS1-2 shRNA interference lentivirus.

Mice were injected either subcutaneously or *via* the caudal vein with K150 cells that had been infected with recombinant lentivirus either overexpressing circTRPS1-2 or shRNA to knock it down. In the subcutaneous neoplasia assay (Fig. 5a), overexpression of circTRPS1-2 led to significantly smaller tumor volumes (Fig. 5b) and weights (Fig. 5c). Knockdown of circTRPS1-2 exerted the opposite effects. In the tumor metastasis assay, knockdown of circTRPS1-2 showed significantly more superficial metastases (Fig. 5d), lung metastases (Fig. 5e, f) and overall metastases at the surface and deep sites (Fig. 5g). Overexpression of circTRPS1-2 showed fewer lung metastases (Fig. 5f) and total metastases (Fig. 5g).

### CircTRPS1-2 may regulate ribosome biogenesis

**Fig. 6:**
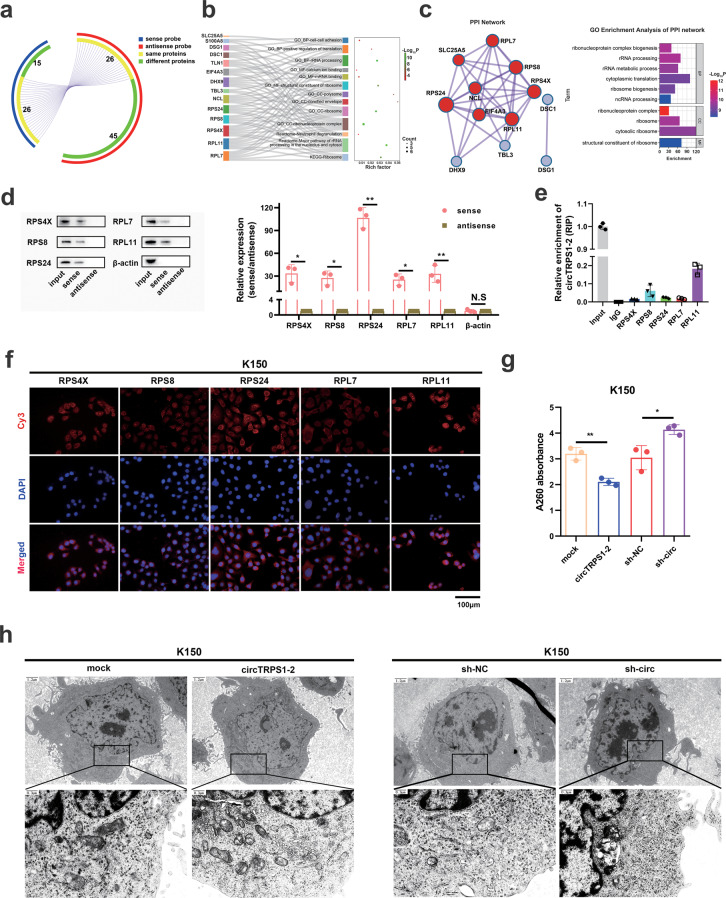
Binding of circTRPS1-2 to ribosomal proteins and effects on ribosome biogenesis. **a** Circos overlap plot showing the number of proteins binding to the sense and antisense probes in the circTRPS1-2 pulldown assay in K150 cells. **b** GO enrichment analysis, Reactome pathway analysis and KEGG pathway enrichment analysis of proteins binding specifically to circTRPS1-2. **c** Interactions among the proteins in panel b. **d** Western blotting against several ribosomal proteins in the RNA precipitate. **e** RIP was performed in K150 cell lysates using antibodies against RPS4X, RPS8, RPS24, RPL7, RPL11 or IgG. Enrichment of circTRPS1-2 was detected by qRT–PCR, relative to circTRPS1-2 expression of input group. Results are from three independent experiments. **f** Immunofluorescence of K150 cells to investigate the subcellular localization of RPS4X, RPS8, RPS24, RPL7 and RPL11. Magnification, 400×. Scale bar, 100 µm. **g** Absorbance (260 nm) of total ribosome extracts from same number of K150 cells over- or underexpressing circTRPS1-2. Results are from three independent experiments. **h** Representative electron micrographs of ribosomes in K150 cells over- or underexpressing circTRPS1-2. The small and black particles in the cytoplasm are ribosomes. Magnification, 20000×. Scale bar (*upper left of images*), 0.3 µm. Results are from three independent experiments. Data are mean ± SD. *P < 0.05, **P < 0.01. circTRPS1-2, circTRPS1-2 overexpression lentivirus; GO, Gene Ontology; KEGG, Kyoto Encyclopedia of Genes and Genomes; mock and sh-NC, negative control lentivirus; RIP, RNA immunoprecipitation; RPS4X, ribosomal protein S4 X-linked; RPS8, ribosomal protein S8; RPS24, ribosomal protein S24; RPL7, ribosomal protein L7; RPL11, ribosomal protein L11; sh-circ, circTRPS1-2 shRNA interference lentivirus.

To clarify the pathway(s) through which circTRPS1-2 may act as a tumor suppressor in ESCC, we immunoprecipitated RNA from K150 cells and identified potential protein binding partners using mass spectrometry (Table S3). After excluding 26 proteins pulled down by both sense and antisense RNA probes, we analyzed 15 proteins that bound specifically to the sense probe (Fig. 6a). In contrast to previous studies [7, 20], we did not detect Argonaute2 in the RNA pulldown assay, suggesting that microRNA sponges might not be the main functional mechanism of circTRPS1-2. Gene Ontology, Reactome pathway and Kyoto Encyclopedia of Genes and Genomes pathway analyses suggested that the 15 proteins are closely related to ribosomes and ribonuclear RNA processing in the nucleolus and cytosol (Fig. 6b, c). We hypothesized that circTRPS1-2 may participate in ribosome biogenesis, so we examined five ribosomal proteins among the 15 candidate interactors: S4X, S8, S24, L7 and L11. We confirmed that circTRPS1-2 binds directly to these proteins using RNA pulldown, followed by western blotting (Fig. 6d, full-length western blots was shown in Original western blots); using RNA immunoprecipitation followed by qRT–PCR (Fig. 6e); and using immunofluorescence staining (Fig. 6f), which localized all five ribosomal proteins to the cytoplasm and nucleus, similar to circTRPS1-2. Next, we infected K150 cells with recombinant lentivirus that overexpressed or knocked down circTRPS1-2. Overexpression led to lower, and knockdown to higher, levels of ribosomes in cellular extracts, based on absorbance at 260 nm (Fig. 6g). Electron microscopy showed that overexpression led to smaller ribosome number and nucleoli, while knockdown had the opposite effects (Fig. 6h).

## Discussion

The progressive elucidation of circRNA functions [11] has made clear their involvement in various cancers [21], and here we provide the first evidence that circTRPS1-2 is significantly downregulated in ESCC. We further show in vitro and in vivo that circTRPS1-2 overexpression suppresses the proliferation and migration of ESCC cells, while circTRPS1-2 knockdown produces the opposite effects. Investigation of circTRPS1-2 interactors suggests that the circular RNA helps regulate ribosome biogenesis. Our study identifies circTRPS1-2 as a target in studies to elucidate how ESCC begins and progresses, and to exploit the RNA as a novel prognostic marker.

We localized circTRPS1-2 to the cytoplasm and nucleus, similar to various ribosomal proteins with which the circular RNA interacted in our experiments and which are known to bind to ribosomal RNAs to form ribosomes [22]. Such interactions may explain the observed effect of circTRPS1-2 on ribosome number [23]. Indeed, circRNAs that accumulate in the nucleus often bind to RNA-binding proteins there, acting as “protein sponges” that influence gene transcription [24]. Many circRNAs that localize to the cytoplasm also function as protein sponges [25], affecting protein expression [26] or distribution [27]. Interestingly, although many circRNAs in the cytoplasm function as microRNA sponges [28], we did not find evidence of such activity for circTRPS1-2 in ESCC cells. Further studies should verify our results and precisely define the function of circTRPS1-2 in healthy and tumor tissue.

CircTRPS1-2 would not be the first circular RNA that regulates ribosome biogenesis. CircANRIL competes with ribosomal RNA for binding to pescadillo homologue 1 protein, impairing the processing of precursor ribosomal RNA for ribosome formation [29]. CircHipk2 inhibits the function or assembly of ribosomes by binding directly to RPL7 [30]. We found that levels of circTRPS1-2 correlated inversely with nucleolus size, which is known to correlate directly with levels of ribosome biogenesis [31] and with the number of ribosomes. These findings are consistent with the idea that abnormally high numbers of ribosomes promote tumor development and progression [32, 33].

We identified a novel circRNA, circTRPS1-2, that appears to be downregulated in ESCC and whose expression may predict prognosis. CircTRPS1-2 may normally exert tumor-suppressive effects by interacting with ribosomal proteins to regulate the production of ribosomes. The present work justifies deeper analysis of circTRPS1-2 in ESCC. Future studies should explore the full range of protein interactors and map the sites of interaction on the circular RNA and its protein partners. The pathways through which these interactions affect ribosome biogenesis should also be elucidated. We will explore circTRPS1-2 as a potential prognostic biomarker and therapeutic target in ESCC.

## Supplementary information

Supplementary Tables

## Data Availability

The datasets used and analyzed during the current study are available from the corresponding author on reasonable request.
