# Supplementary material for: Circular RNA circTRPS1-2 inhibits the proliferation and migration of esophageal squamous cell carcinoma by reducing the production of ribosomes
Source: Cell Death Discov. 2023 Jan 12;9:5. doi: 10.1038/s41420-023-01300-9 (PMC9837173; doi:10.1038/s41420-023-01300-9)
Supplement: Supplementary file 1 — Supplementary Tables [file 41420_2023_1300_MOESM1_ESM.docx]

| **Table S1. Sequences of primer, probe, siRNA and shRNA** | | |
| --- | --- | --- |
| **Name** | **sequence** | |
| hsa_circ_0006156 | Forward primer | 5’-AAGAAAACAGAGCGACGAGCA-3’ |
|  | Reverse primer | 5’-GGGTAGCТTGGGGGATAACAC-3’ |
| hsa_circ_0003764 | Forward primer | 5’-GTGAACТGGCCAGACCATGA-3’ |
|  | Reverse primer | 5’-CAAAGGCCAATAGCGCТCAC-3’ |
| hsa_circ_0000198 | Forward primer | 5’-TGCТGTGТCТGGGATAACCТC-3’ |
|  | Reverse primer | 5’-CCAGAAGACGGATCТTGACТCТ-3’ |
| hsa_circ_0007324 | Forward primer | 5’-TCGCAGCCATTGAGGGTAATC-3’ |
|  | Reverse primer | 5’-AGGTGТCCAGTTTТCТTGТCТ-3’ |
| hsa_circ_0016600 | Forward primer | 5’-ACCTACTTTGGAATGGCTATCAGAA-3’ |
|  | Reverse primer | 5’-ТGGТТТССТССТТGТССАТСТ-3’ |
| hsa_circ_0026782 | Forward primer | 5’-GAAGTТCТGTGAGGGACGCC-3’ |
|  | Reverse primer | 5’-CACAAAGCAGCGACCAATCA-3’ |
| hsa_circ_0085362 | Forward primer | 5’-TGGCGGAGAGAAGTCTGG-3’ |
|  | Reverse primer | 5’-GGCATGTGGCTTTAGAGTGC-3’ |
| hsa_circ_0078299 | Forward primer | 5’-GCCТCAGGAGGCTTAACCAA-3’ |
|  | Reverse primer | 5’-СТСТСТGТССААСТGСАТGАТ-3’ |
| hsa_circ_0000099 | Forward primer | 5’-AAGGTTGGGGTTTCATGCCТ-3’ |
|  | Reverse primer | 5’-AGTTGAGTGATGTGCCAGGAA-3’ |
| hsa_circ_0008967 | Forward primer | 5’-AGGATGTTТGGATCТGGCCG-3’ |
|  | Reverse primer | 5’-ТCAACATGGTGCТGТCGTGT-3’ |
| hsa_circ_0001546 | Forward primer | 5’-CТTCCCТTGGAATCCCТGGТC-3’ |
|  | Reverse primer | 5’-GGCТGCТTCAGTTAGCAGTG-3’ |
| hsa_circ_0001882 | Forward primer | 5’-GCCТCAGCTAGTTCCТCGAC-3’ |
|  | Reverse primer | 5’-GCAGCACCCТТCCTAGAGTT-3’ |
| hsa_circ_0001073 | Forward primer | 5’-AGATGGAAGTCACACAGCCC-3’ |
|  | Reverse primer | 5’-AGGTAGCAAAACAATGCCGC-3’ |
| hsa_circ_0000638 | Forward primer | 5’-GCТGGCTTTGTTCCCAATGA-3’ |
|  | Reverse primer | 5’-TAAGCAGGACACТTCACCТCC-3’ |
| hsa_circ_0000117 | Forward primer | 5’-TGGAAGTTCACAAATGGGTGC-3’ |
|  | Reverse primer | 5’-GGTTGGGAATAGGGACТGGTG-3’ |
| TRPS1 | Forward primer | 5’-CGGTGAGCAGATTATTAGGAGGAG-3’ |
|  | Reverse primer | 5’-CTCTCTAACGGGCTTCCATTGA-3’ |
| β-actin | Forward primer | 5’-TGGCACCCAGCACAATGAA-3’ |
|  | Reverse primer | 5’-CTAAGTCATAGTCCGCCTAGAAGCA-3’ |
| GAPDH | Forward primer | 5’-GCACCGTCAAGGCTGAGAAC-3’ |
|  | Reverse primer | 5’-TGGTGAAGACGCCAGTGGA-3’ |
| U1 snRNA |  | Millipore, Cat. #CS203215 |
| circTRPS1-2 | FISH probe | 5’-GATCTGTACATCCGTAACAGGGACTGG-3’ |
| si-circ#1 |  | 5’-TGTTACGGATGTACAGATC-3’ |
| si-circ#2 |  | 5’-AGTCCCTGTTACGGATGTA-3’ |
| sh-circ |  | 5’-TGTTACGGATGTACAGATC-3’ |
| sense probe | RNA pulldown probe | 5’-GUCCCUGUUACGGAUGUACAGAUCA-3’ |
| antisense probe | RNA pulldown probe | 5’-UGAUCUGUACAUCCGUAACAGGGAC-3’ |

| **Table S2.** Clinicopathological features of 30 ESCC patients and the expressions of circTRPS1-2 | | | | | | |
| --- | --- | --- | --- | --- | --- | --- |
| **Parameters** |  | **Group** | **Cases** | **circTRPS1-2 expression** | | |
|  |  |  |  | **High** | **Low** | ***P* value** |
| Gender |  | Male | 28 | 13 | 15 | - |
|  |  | Female | 2 | 2 | 0 |  |
| Age at surgery |  | <65 | 17 | 10 | 7 | 0.462 |
|  |  | ≥65 | 13 | 5 | 8 |  |
| T stage |  | pT1-T2 | 6 | 5 | 1 | 0.169 |
|  |  | pT3-T4 | 24 | 10 | 14 |  |
| Lymph node metastasis |  | Absent | 12 | 7 | 5 | 0.710 |
|  |  | Present | 18 | 8 | 10 |  |
| TNM stage |  | Ⅰ-Ⅱ | 9 | 7 | 2 | 0.109 |
|  |  | Ⅲ-Ⅳ | 21 | 8 | 13 |  |
| Total |  |  | 30 |  |  |  |
| *P* < 0.05 represents statistical significance (Fisher’s exact test) | | | | | | |

| **Table S3.** Proteins binding to circTRPS1-2 | | | |  |
| --- | --- | --- | --- | --- |
| **Protein IDs** | **Species** | **Gene Name** | |  |
| P63261 | Homo sapiens | | actin gamma 1 (ACTG1) |  |
| D6RA73 | Homo sapiens | | heat shock protein family A (Hsp70) member 9 (HSPA9) |  |
| P81605 | Homo sapiens | | dermcidin (DCD) |  |
| J3QSA3 | Homo sapiens | | ubiquitin B (UBB) |  |
| A0A0U1RQJ0 | Homo sapiens | | DEAD-box helicase 17 (DDX17) |  |
| E9PKZ0 | Homo sapiens | | ribosomal protein L8 (RPL8) |  |
| E5RGW4 | Homo sapiens | | nucleophosmin (NPM1) |  |
| Q5T8U3 | Homo sapiens | | ribosomal protein L7a (RPL7A) |  |
| P01040 | Homo sapiens | | cystatin A (CSTA) |  |
| F8WD59 | Homo sapiens | | ribosomal protein SA (RPSA) |  |
| Q9BQE3 | Homo sapiens | | tubulin alpha 1c (TUBA1C) |  |
| P26373 | Homo sapiens | | ribosomal protein L13 (RPL13) |  |
| A0A087WVQ9 | Homo sapiens | | eukaryotic translation elongation factor 1 alpha 1 (EEF1A1) | |
| A2A3R5 | Homo sapiens | | ribosomal protein S6 (RPS6) |  |
| E9PPU1 | Homo sapiens | | ribosomal protein S3 (RPS3) |  |
| Q01469 | Homo sapiens | | fatty acid binding protein 5 (FABP5) |  |
| F5H1T5 | Homo sapiens | | CAP-Gly domain containing linker protein 1 (CLIP1) |  |
| B5MCP9 | Homo sapiens | | ribosomal protein S7 (RPS7) |  |
| M0QZU1 | Homo sapiens | | ribosomal protein L13a (RPL13A) |  |
| P61626 | Homo sapiens | | lysozyme (LYZ) |  |
| M0R019 | Homo sapiens | | heterogeneous nuclear ribonucleoprotein M (HNRNPM) |  |
| P11142 | Homo sapiens | | heat shock protein family A (Hsp70) member 8 (HSPA8) |  |
| F8VUA6 | Homo sapiens | | ribosomal protein L18 (RPL18) |  |
| P04406 | Homo sapiens | | glyceraldehyde-3-phosphate dehydrogenase (GAPDH) |  |
| A0A1X7SBS1 | Homo sapiens | | heterogeneous nuclear ribonucleoprotein U (HNRNPU) |  |
| P26641 | Homo sapiens | | eukaryotic translation elongation factor 1 gamma (EEF1G) |  |
| E7ETK0 | Homo sapiens | | ribosomal protein S24 (RPS24) |  |
| G3V2N7 | Homo sapiens | | ring finger protein 212B (RNF212B) |  |
| H3BN88 | Homo sapiens | | transducin beta like 3 (TBL3) |  |
| I3L3H2 | Homo sapiens | | eukaryotic translation initiation factor 4A3 (EIF4A3) |  |
| P05109 | Homo sapiens | | S100 calcium binding protein A8 (S100A8) |  |
| P05141 | Homo sapiens | | solute carrier family 25 member 5 (SLC25A5) |  |
| P18124 | Homo sapiens | | ribosomal protein L7 (RPL7) |  |
| P19338 | Homo sapiens | | nucleolin (NCL) |  |
| P62701 | Homo sapiens | | ribosomal protein S4, X-linked (RPS4X) |  |
| Q02413 | Homo sapiens | | desmoglein 1 (DSG1) |  |
| Q08211 | Homo sapiens | | DExH-box helicase 9 (DHX9) |  |
| Q08554 | Homo sapiens | | desmocollin 1 (DSC1) |  |
| Q5JR95 | Homo sapiens | | ribosomal protein S8 (RPS8) |  |
| Q5VVC8 | Homo sapiens | | ribosomal protein L11 (RPL11) |  |
| Q9Y490 | Homo sapiens | | talin 1 (TLN1) |  |
